# The 4D Space-Time Dimensions of Facial Perception

**DOI:** 10.3389/fpsyg.2020.01842

**Published:** 2020-07-28

**Authors:** Adelaide L. Burt, David P. Crewther

**Affiliations:** Centre for Human Psychopharmacology, Swinburne University of Technology, Melbourne, VIC, Australia

**Keywords:** 4D, four-dimensional, 3D, three-dimensional, faces, perception, psychology, neuroscience

## Abstract

Facial information is a powerful channel for human-to-human communication. Characteristically, faces can be defined as biological objects that are four-dimensional (4D) patterns, whereby they have concurrently a spatial structure and surface as well as temporal dynamics. The spatial characteristics of facial objects contain a volume and surface in three dimensions (3D), namely breadth, height and importantly, depth. The temporal properties of facial objects are defined by how a 3D facial structure and surface evolves dynamically over time; where time is referred to as the fourth dimension (4D). Our entire perception of another’s face, whether it be social, affective or cognitive perceptions, is therefore built on a combination of 3D and 4D visual cues. Counterintuitively, over the past few decades of experimental research in psychology, facial stimuli have largely been captured, reproduced and presented to participants with two dimensions (2D), while remaining largely static. The following review aims to advance and update facial researchers, on the recent revolution in computer-generated, realistic 4D facial models produced from real-life human subjects. We delve in-depth to summarize recent studies which have utilized facial stimuli that possess 3D structural and surface cues (geometry, surface and depth) and 4D temporal cues (3D structure + dynamic viewpoint and movement). In sum, we have found that higher-order perceptions such as identity, gender, ethnicity, emotion and personality, are critically influenced by 4D characteristics. In future, it is recommended that facial stimuli incorporate the 4D space-time perspective with the proposed time-resolved methods.

## The 4D Space-Time Perspective of Facial Perception

What does our visual-perceptual system “*see*” when we view a face? Prima facie, we see a *three-dimensional* form, evolving over time, that enables multifaceted social, cognitive and affective perceptions of one another. From cognitive information such as identity and recognition; to detecting complex patterns of speech or emotion; to gleaning important social cues such as gender, ethnicity, age or health. The human face has been defined by other authors as a multi-dimensional pattern which evolves over time (Zhang et al., 2003; Liu et al., 2012; Marcolin and Vezzetti, 2017). This multi-dimensional, complex pattern is also highly individualized; varying from person to person. Thus, each human face possesses concurrently a unique volumetric structure and surface pattern in three dimensions (or 3D) and a temporal pattern across time in four dimensions (or 4D). The 3D volumetric structure or form of human facial features contains spatial dimensions of breadth, height and width, combined with a unique surface pattern. The 4D temporal pattern of the human face encompasses all dynamic movement and changes to this 3D spatial form that evolve with time. Little is known however, about how we detect and perceive the 3D spatial or 4D temporal patterns of human faces on two-dimensional computer screens.

Indeed, over the past few decades, the field of facial perception has been largely built upon 2D static images and photographs displayed on screens, which are computationally reproduced with two-dimensions of width and height (for a review see Matsumoto, 1988; Lundqvist et al., 1998; Beaupré et al., 2000; Tottenham et al., 2009; Langner et al., 2010; Krumhuber et al., 2017). 2D dynamic or video-recorded stimuli have likewise made a valuable contribution; demonstrating facial recognition may be enhanced with motion (Kanade et al., 2000; Wallhoff, 2004; Pantic et al., 2005; Lucey et al., 2010; Krumhuber et al., 2013, 2017; Dobs et al., 2018) although this issue remains unresolved (Christie and Bruce, 1998; Krumhuber et al., 2013, 2017). 2D stimuli demonstrate robust and reliable human facial recognition performance across behavioral studies of face perception (Beaupré et al., 2000; Goeleven et al., 2008; Rossion and Michel, 2018). These stimuli have helped characterize individual abilities in facial recognition- from face experts (Tanaka, 2001; Russell et al., 2009) to describing a continuum of individual differences in face recognition abilities (Wilmer et al., 2010; Dennett et al., 2012; Rhodes et al., 2014); and to describe the challenges of face perception exhibited in clinical or atypical populations (Behrmann and Avidan, 2005; Halliday et al., 2014). Some of the most influential neuroimaging models of facial processing have employed 2D faces, discovering spatio-temporal networks and regions of activation (Haxby et al., 2000; O’Toole et al., 2002; Kanwisher and Yovel, 2006). In addition, significant practical advantages are afforded by these stimuli such as; the ability to more precisely control and manipulate stimuli; reduced data size and reduced computational load or complexity (Wallbott and Scherer, 1986; Motley and Camden, 1988; Bruce et al., 1993; Russell, 1994; Elfenbein and Ambady, 2002). Overall, 2D faces are a valuable source of experimental stimuli as they offer a robust level of control, reproducibility and replication.

In recent years, three-dimensional virtual representations of the human face that are more concurrent with our everyday social experiences, are beginning to be recognized as essential stimuli (Blauch and Behrmann, 2019; Zhan et al., 2019). While these newer technologies are not being utilized in psychology, they offer several advantages as naturalistic, ecologically valid representations of human faces. Historically however, creating a simulation of a biological human face on a two-dimensional computer screen in 3D and 4D formats, has proven difficult. Simulating concurrently the unique 3D structure of a face, with the added dimension of movement over time, has made computational recovery a challenging field. More recently, computer science has seen rapid advancement in facial reproduction technology in 3D and 4D formats, enabled through development within gaming and virtual-reality (Blascovich et al., 2002; Bailenson et al., 2004; Bülthoff et al., 2018; Chen et al., 2018; Smith and Neff, 2018). In fact, computer-generated objects, human bodies and faces give us a new sense of virtual dynamics and interaction, producing the visual illusion of being able to be moved, rotated or grasped in a virtual space (Freud et al., 2018a; Chessa et al., 2019; Mendes et al., 2018; Sasaoka et al., 2019). Evidence is beginning to emerge that it is also critical to actively explore 3D spaces when viewing faces (Bailenson et al., 2004; Bülthoff et al., 2018). Thus, while in the past we have only been able to experiment with facial perception from a largely two-dimensional or static perspective, our overarching goal is to open-up other dimensions of these newer stimuli for researchers. It is likely by viewing human facial modeling from a space-time (4D) perspective, that much will be learned about how naturalistic and ecologically valid facial perception is performed in our interactions with each other.

## How are 3D and 4D Facial Databases Captured and Reproduced Into a Digital Stimulus?

How do we make *you* *really* look like *you*on a digital screen? Facial databases, comprise sets of facial stimuli which capture and reproduce multiple human subjects or individuals into a digital format. Virtual humans, often termed “virtual busts or avatars,” represent a burgeoning area of computational development (Blascovich et al., 2002; Bailenson et al., 2004). While “virtual avatars” are being presented to the viewer on a two-dimensional flat screen, they elicit the illusion of being three-dimensional. These virtual computer-generated human bodies possess impressive anatomical-accuracy, including of both individual body parts and holistic bodies (Allen et al., 2003; Cofer et al., 2010; Ramakrishna et al., 2012; Pujades et al., 2019). Likewise, the reproduction of biological human faces into a virtual model on-screen, has been achieving a sense of realism through the extensive development of several teams (Wang et al., 2004; Vlasic, 2005; Gong et al., 2009; Weise et al., 2009; Sandbach et al., 2012; Bouaziz et al., 2013; Chen et al., 2013; Garrido et al., 2013; Li et al., 2013; Shi et al., 2014; Suwajanakorn et al., 2014; Zhang et al., 2014; Cao et al., 2015; Hsieh et al., 2015; Thies, 2015; Saito et al., 2016; Jeni et al., 2017; Zollhöfer et al., 2018). Digitized facial databases are now available as stimuli across several media formats; including 3D static models or images (Yin et al., 2006; Saito et al., 2016) 4D dynamic movies or videos (Wang et al., 2004; Vlasic, 2005; Gong et al., 2009; Weise et al., 2009; Garrido et al., 2013; Shi et al., 2014; Suwajanakorn et al., 2014; Zhang et al., 2014; Cao et al., 2015; Thies, 2015; Jeni et al., 2017) and as 3D/4D interactive stimuli, such as in virtual reality (Bouaziz et al., 2013) or with eye-gaze tracking (Thies, 2017; Chen et al., 2018).

**FIGURE 1:**
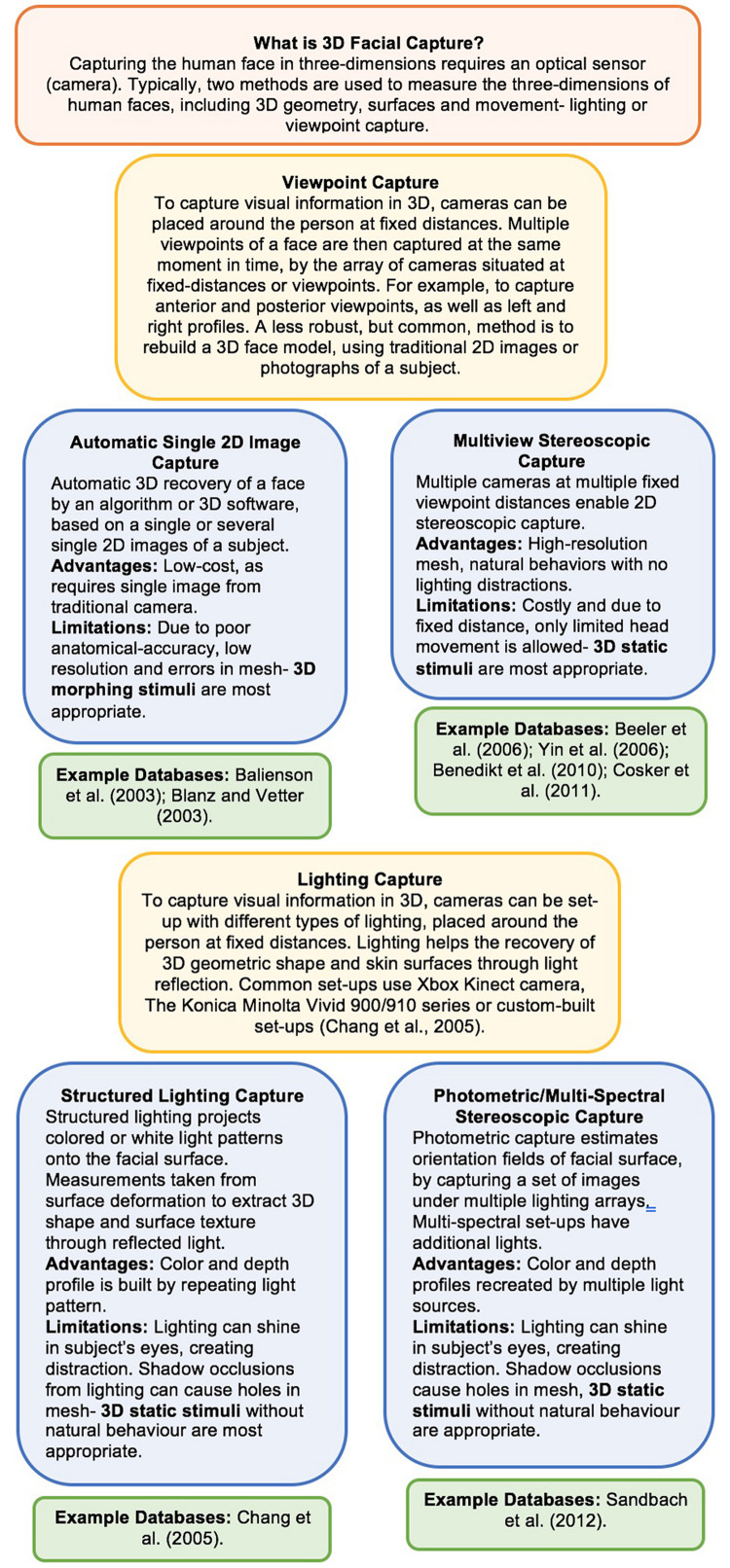
Techniques of lighting and viewpoint capture used in highly cited 3D + 4D facial databases. See comprehensive review of capture techniques by Sandbach et al. (2012) and Table 1 in Zollhöfer et al. (2018).

The capture techniques, reconstruction algorithms and 3D software packages used to develop and display a life-like 3D or 4D human face into a virtual form is a highly individualistic, complex process and is achieved differently by each database. Thus, the different capture and reproduction techniques are comprehensively surveyed and reviewed elsewhere (Gaeta et al., 2006; Beeler et al., 2010; Sandbach et al., 2012; Patil et al., 2015; Feng and Kittler, 2018; Zollhöfer et al., 2018). Briefly however, the databases above demonstrate methodologically how to produce an anatomical head model through mesh, polygon or geometric structures, using width x height x depth coordinates (Egger et al., 2019). They demonstrate the unique and highly technical challenge of producing surface rendering or skin rendering with shape deformations that are anatomically precise, while capturing environmental lighting, reflectance, pigmentation, shadow and texture (Egger et al., 2019). The 4D databases also exhibit how animation and modeling are combined to produce anatomically accurate rigid and non-rigid head and facial movements, whilst maintaining 3D shape and surface properties (Egger et al., 2019). For a more detailed overview of how facial information is captured with lighting or viewpoint camera arrays within these databases, see Figure 1. To see an illustration of the workflow process of 3D modeling, rendering and animation for human faces and its level of complexity and limitations, see Marschner et al. (2000), Murphy and Leopold (2019) for primate faces. To review state-of-the-art advancements over the past 20 of development, as well as the more technical challenges that remain in the field producing 3D animated faces see the comprehensive review by Egger et al. (2019).

**FIGURE 2:**
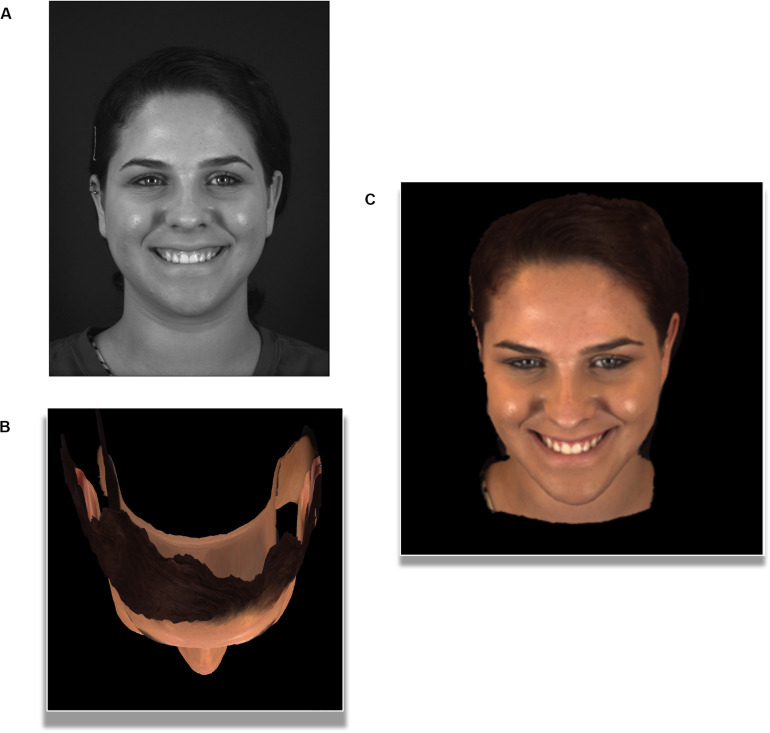
2D image compared to the 4D video-frame of a highly cited 4D facial database (The images pictured above were reproduced, modified and adapted from the facial database originally developed by Zhang et al. (2013; 2014). Copyright 2013–2017, The Research Foundation for the State University of New York and University of Pittsburgh of the Commonwealth System of Higher Education. All Right Reserved. BP4D-Spontaneous Database, as of: 26/03/2018. **(A)** A flattened face. Traditional camera set-up produces a 2D image or photograph in gray-scale. **(B)** A bird’s eye view of 3D geometric mesh. Multi-view camera set-up producing a 3D structured facial mesh, with skin texture, using 30,000–50,000 vertices (viewed from top of skull). **(C)** A 3D sense of re-created depth. Despite being on a two-dimensional computer screen, a 3D face reconstructed with multi-view camera set-up, produced an illusion of depth. The final reconstruction produces the same facial model, which stands out from the background and appear more lifelike than the 2D photograph. When working with 3D facial stimuli, we can change our viewpoint of the facial stimulus. Here we have manipulated the viewer angle of the camera along a *Z*-axis is possible (top-frontal view), to view the same frame of a face from many possible 360 degree viewpoints.

Of note, is that the reproduction of 3D facial models still present challenges in stimuli recreation (Marschner et al., 2000; Egger et al., 2019). Further challenges in implementing 3D or 4D facial models are also unique to research fields such as psychology and neuroscience. Faces are a unique, special class of images in human vision, where deviations from anatomically accurate geometry, surfaces or movement can be described as incorrect or “uncanny” by human observers (Marschner et al., 2000; Kätsyri, 2006; Seyama and Nagayama, 2007). Therefore, participant viewing conditions must be well-controlled to ensure models are presented at the same viewing distance and settings (e.g., environment lighting), as original development (Marschner et al., 2000). In addition, future development of 3D or 4D databases for experimental designs, can further improve stimuli by being built as full-volumes and not surfaces. For example, the database presented in Figure 2B, experiences a loss of data from the posterior viewpoint of the head, which may impede studies examining VR environments (Bulthoff and Edelman, 1992; Stolz et al., 2019; Lamberti et al., 2020) or multiple viewpoints of a facial stimulus (Bülthoff et al., 2018; Abudarham et al., 2019; Zhan et al., 2019). Overall, despite some remaining challenges, 3D and 4D facial capture and reconstruction has demonstrated that the unique facial features belonging to you and me, such as the cheeks, eyes and nose, can simulated on-screen in a highly realistic manner (see Figure 2).

## How Popular are 3D and 4D Facial Stimuli?

**FIGURE 3:**
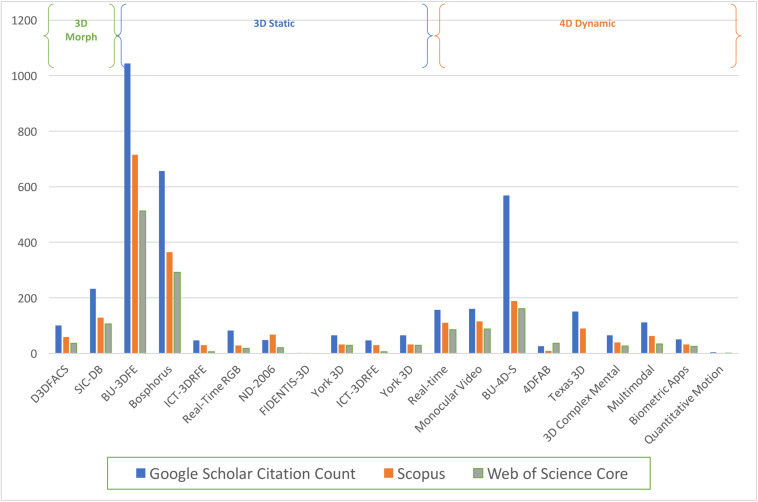
3D and 4D facial databases publication frequency across Google Scholar, Scopus and Web of Science. Search on 13/02/2020, including count of the original published journal article of each database.

An initial quantification of the most widely cited 3D stimuli, suggests the static 3D facial database *BU-3DFE* by Yin et al. (2006) and the Bosphorus *3D* database by Savran et al. (2008) are at the forefront. Similarly, the dynamic video-recorded BU4D-S 4D database produced by Zhang et al. (2013, 2014) is the leading 4D database in citation count (Figure 3). It is worth highlighting that a selection of 3D and 4D facial databases, have been developed with specific applications across disciplines. Facial identification and recognition studies, commonly referred to as biometrics, may benefit from the databases developed by Benedikt et al. (2010); Feng et al. (2014), and Zhang and Fisher (2019). A relatively large corpus of datasets has been developed to present stimuli as three-dimensional forms or structures on-screen (built with breadth, height and depth), as static emotional expressions (*FIDENTIS-3D*; Urbanová et al., 2018; *Bosphorus*; Savran et al., 2008; *BU-3DFE*; Yin et al., 2006; *ICT-3DRFE*; Stratou et al., 2011; *ND-2006*; Faltemier et al., 2008; *Casia 3D*; Zhong et al., 2007; *The York 3D*; Heseltine et al., 2008; *GAVDB*; Moreno, 2004; *Texas 3DFRD*; Gupta et al., 2010). To establish 4D dynamical facial stimuli, faces have also been video-recorded and re-built with naturalistic spontaneous or acted emotions; as dynamical 4D objects that produce movement over a series of frames (Gur et al., 2002; *Cam3D*; Mahmoud et al., 2011; *BU-4DFE*; Yin et al., 2006; *BU4D-S*; Zhang et al., 2014; 4DFAB; Cheng et al., 2018). Studies of facial perceptions which require precise control and manipulation of artificial motion properties, may benefit from the databases developed for morphing paradigms (*D3DFACS*; Cosker et al., 2011; *SIC DB*; Beumier and Acheroy, 1999). In sum, more extensive surveys of 3D and 4D facial databases are available; whereby facial researchers can select stimuli from a range of available databases (*for a review see* Weber et al., 2018; Zhou and Xiao, 2018; Sadhya and Singh, 2019; Sandbach et al., 2012; Smeets et al., 2012). For a comprehensive survey of the publicly facial databases with both 2D and 3D stimuli, see Sadhya and Singh (2019).

**FIGURE 4:**
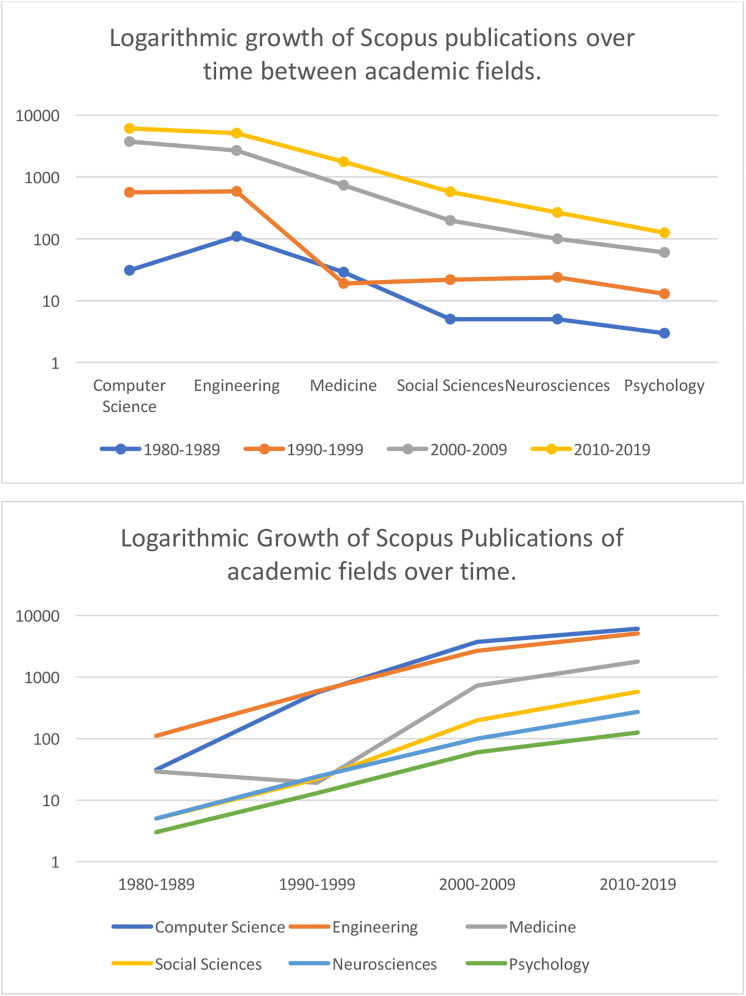
Logarithmic Scale of Scopus publication growth from 1980 to 2019 across fields containing key search terms. Search terms: (“allintitle, abstract or keywords”: “3-D face” OR “3D face” OR “three-dimensional face” OR “three-dimensional face” OR “4D”).

**FIGURE 5:**
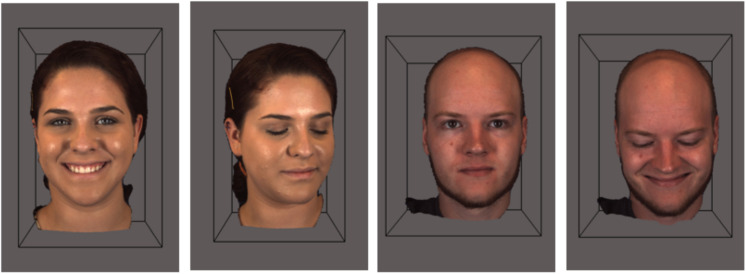
BP-4D-S facial database presented on gray background with bounding box representing a 3D depth plane (The images pictured above were reproduced, modified and adapted from the facial database originally developed by Zhang et al. (2013, 2014). Copyright 2013–2017, The Research Foundation for the State University of New York and University of Pittsburgh of the Commonwealth System of Higher Education. All Right Reserved. BP4D-Spontaneous Database, as of: 26/03/2018. 3D faces are built on-screen as models with a geometric mesh in a three-dimensional plane; presenting an anatomically accurate modeled object with width, height and depth. This is captured by the 3D camera set-up. This reconstruction by computer-scientists is highly individualized, but is typically underlined by a geometric mesh or structure. The 3D surface features of the individual are then rendered. As the face moves around in 3D space, it becomes apparent why having three dimensions maintains facial structure more realistically than a facial model built with only two dimensions, as indicated by the depth plane.

At first glance, the citation count pictured in Figure 4 appears impressive. Yet, we observed that this citation count is largely driven by the development of computer science and engineering; including capturing, reproducing, recognition and tracking of 3D and 4D facial technology. Thus we examined logarithmic growth across academic fields below (Figure 5). Notably psychology and neuroscience articles associated with terms such as “3D or 4D faces,” in titles, abstracts and keywords, demonstrate reduced logarithmic growth compared to other academic fields. It appears that psychologists and neuroscientists continue to reference more traditional, two-dimensional facial databases in their research. Although not exhaustive, some examples of the most popular, highly cited repositories of 2D image-based facial sets across psychology include; “*The Montreal Set of Face Displays*,” (Beaupré et al., 2000) “*The Radboud Face Set*,” (Langner et al., 2010) “*The Karolinska Directed Faces*,” (Lundqvist et al., 1998) “*The Japanese-Caucasian Facial Affect Set*,” (Matsumoto, 1988) and “*The NimStim Set of Facial Expressio*ns,” (Tottenham et al., 2009). More recently, standardized stimulus sets of 2D video-recorded faces have been developed; the “*Cohn-Kanade (CK)*,” (Kanade et al., 2000) “*The Extended Cohn-Kanade (CK+) Dataset*,” (Lucey et al., 2010) “*Face and Recognition Network-Group*,” (Wallhoff, 2004) “*MMI face database*,” (Pantic et al., 2005). For a comparative review of the most-cited 2D static and dynamic face stimuli utilized by the research community, please see the review provided by Krumhuber et al. (2017).

Recent experimental studies utilizing 4D dynamical faces, however, have raised issue with whether the human face should be reduced on-screen to a 2D or static pattern (Elfenbein and Ambady, 2002). While these arguments have existed for a lengthy period within the literature, the recent cultural shift in gaming and virtual-reality has enabled us to better envision “*virtual-facial realism*.” It is timely, therefore, to review what 3D and 4D dynamic face stimuli can offer by opening other dimensions of facial stimuli for researchers in all fields.

## What is the 3D Spatial Pattern of the Human Face?

### The Influence of 3D Volume and Surfaces in Building Facial Perception

Despite experimental stimuli being displayed on a two-dimensional, flat screen such as a computer monitor, the human visual system perceives 3D structure remarkably efficiently. Three-dimensional objects or models rely upon a geometric structural and surface model presenting accurate reconstruction of breadth, height and *importantly depth*, while still being presented on a two-dimensional screen (Hallinan et al., 1999; Marschner et al., 2000; Egger et al., 2019). Representing detailed facial tissue, bone structure and the musculature system, which make up the human face, has not been an easy feat, yet is now computationally achievable (Zhang et al., 2003; Ekrami et al., 2018). Moreover, these 3D facial models are revealing key differences about both individual recognition, as well as categorization or classification of human faces as objects.

To disseminate which visual elements of 3D geometry contribute to facial recognition in humans, psychophysical studies have begun to examine volume and curvilinear shape (Hallinan et al., 1999; Chelnokova and Laeng, 2011; Jones et al., 2012; Eng et al., 2017). Psychophysical research comparing 2D and stereoscopic 3D face stimuli are revealing some potential key differences in how we detect and perceive 3D models. Stereoscopic stimuli are used to develop 3D faces from 2D images paired with red-cyan stereoscopic glasses, which elicit depth through binocular disparity cues (Schwaninger and Yang, 2011; Häkkinen et al., 2008; Lambooij et al., 2011). Using these methods, Chelnokova and Laeng (2011) have interpreted the increased fixation time spent on cheeks and noses observed in their study during the 3D viewing condition, as a preference for the more volumetric properties of faces. In the case of unfamiliar faces, recognition of identities were performed better with upright 3D stereoscopic faces, compared to matched 2D stimuli (Chelnokova and Laeng, 2011; Eng et al., 2017). Comparatively, inverted faces elicited no difference in identity recognition between 2D or 3D stereoscopic form, suggesting this is an effect observed using a holistic face template (Eng et al., 2017). Further, reaction times appear significantly faster for identifying 3D upright compared to 3D inverted faces, suggesting it is more efficient to perceive upright faces (Eng et al., 2017). Reaction times to 3D stereoscopic stimuli however, appear to be generally slower than 2D matched-stimuli (Chelnokova and Laeng, 2011; Eng et al., 2017). Thus, these preliminary studies present initial psychophysical evidence that upright 3D stereoscopic information contained within a face, may provide better accuracy in identification than a matched 2D image. Albeit, with a slower response. The implications of these studies suggest that the structural facial features associated with 3D geometric volume, such as cheeks and noses, may add a critical element during naturalistic facial processing. Overall, these studies provide preliminary psychophysical evidence that stereoscopic volume within facial features may be involved in perception; however, with large standard errors more rigorous investigation is required.

Further studies have explored how we perceive 3D faces with a volumetric structure, through our classification of their curvilinear properties or organic shape. Curvilinear geometry is defined as a shape bound by curved, organic lines as seen in animate objects. Inanimate objects, characteristically include linear geometry or edges bound by straight lines. In fact, facial identity or individuality is often built into 3D computerized models, by using multiple, individualized curves, rather than the straight lines used to build objects (Yin et al., 2006; Liu et al., 2012; Li et al., 2018). Researchers have likewise utilized curvilinear shapes in 2D images to demonstrate that the entire class of animate objects (e.g., faces, bodies, animals) are detected and classified principally through curvilinear discrimination (Levin et al., 2001; Long et al., 2016; Zachariou et al., 2018). Inanimate object recognition, by comparison, relies mainly on detecting rectilinear shapes and lines, such as that observed in a box or house object (Levin et al., 2001; Long et al., 2016; Zachariou et al., 2018). In terms of human 3D perception of faces, Jones et al. (2012) have demonstrated that we can perceive some personality traits in others, using exclusively 3D shape or curvilinearity. Presenting 3D facial scans exclusively with curvilinear shape, with all surface properties removed, participants accurately reported above-chance another person’s self-reported Big-5 trait-level of “Neuroticism” (Goldberg, 1993; Jones et al., 2012). Thus, curvilinear shape appears to be a visual cue that aids perception of faces, as well as categorizing social or personality traits in others. Although, overall to identify traits, a combination of geometry and surface cues have been found to enhance perception (Jones et al., 2012).

Alongside, a geometric volume or structure, 3D surface rendering is a key process in developing 3D and 4D facial models (*human faces see* Marschner et al., 2000 *or for primate faces* Murphy and Leopold, 2019). The surface of the human face, or epogeneous skin layer, is a highly individualized, changeable feature which aids recognition. Our unique skin surface, including our individualized texture, shading, illumination, reflectance and pigmentation can be reproduced and rendered on a computer screen by using a 3D facial model (Egger et al., 2019). One common skin rendering technique used in these databases, for example, is bump surface models, with anatomically correct models being the ultimate goal (Yin et al., 2006; Zhang et al., 2014). As early as 1991, the psychological or neuroscientific literature was described as “surface primitive” (Bruce et al., 2013) indicating it lacked evidence of how 3D rendered surfaces contributed to our perceptions. Since then 2D facial imagery has helped reveal the contributions of surfaces to perception; where our recognition of faces becomes impaired due to textural or color cues being unexpected, such as in contrast polarity (Hayes et al., 1986; Liu and Chaudhuri, 1997, 2003; Itier and Taylor, 2002; Russell et al., 2006), or when lighting direction is in an unusual or bottom position (Hill and Bruce, 1996; Braje et al., 1998; Johnston et al., 2013).

Similarly, surface properties conveyed by 3D rendering have shown that several factors contribute to our perceptions, such as texture gradients, reflectance, pigmentation and lighting styles. In viewing 3D images, both surface and structure have been considered as key components of our perception of unfamiliar faces or strangers (O’Toole et al., 2005). When examining 3D scanned models of unfamiliar faces, Liu et al. (2005) reveal texture gradients alone can facilitate recovery of facial identity above-chance, although the performance was poorer than shape-from-shading, suggesting shadows and illumination may be more critical. More recently it has been suggested that when perceiving familiar 3D faces of friends and acquaintances, we do not heavily rely on texture or surface information and instead rely upon 3D structure (Blauch and Behrmann, 2019). Contrastingly, when looking at 2D faces, familiar faces have been suggested to be recognized more so from an individuals’ surface information, rather than the two-dimensional shape (Russell and Sinha, 2007). For example, where skin surfaces are removed from an underlying 2D facial shape, familiar faces cannot be recognized (Russell and Sinha, 2007).

As well as texture, 3D rendering also encompasses coloring or skin pigmentation, which has been shown to be a key feature of identification (Yip and Sinha, 2002; Russell et al., 2007). One study of the effect of color and pigmentation cues on 3D face models, describes our accurate ability to detect kinship or genetic relationships of individuals we have not seen previously (Fasolt et al., 2019). For example, we can accurately detect kinship through skin pigmentations exclusively, as well as genetic facial morphology. Interestingly, Fasolt et al. (2019) concluded that we did not require a combination of structure and pigmentation, as high-accuracy was obtained from each of these properties alone. Similarly, physical health is perceived in another, based on skin pigmentation and texture, more so than 3D shape. For example, Jones et al. (2012) demonstrate physical health of a 3D face can be accurately classified using texture exclusively, based on true health in daily living (Jones et al., 2012). Skin surfaces were also suggested to provide accurate information about a person’s Big-5 self-reported trait levels of “Agreeableness” and “Extraversion” (Jones et al., 2012).

In summary, it appears the unique, complex geometric forms and surface properties contained within 3D faces, may be a *vital factor* underlying our perceptions of each other. This effect has been investigated in identity recognition tasks of unfamiliar faces (Levin et al., 2001; Pizlo et al., 2010; Long et al., 2016; Eng et al., 2017; Zachariou et al., 2018), memory of familiar faces (Blauch and Behrmann, 2019; Kinect Xbo,, 2019; Zhan et al., 2019) and our detection of important social cues, such as genetic relatedness (Fasolt et al., 2019) health or personality traits (Jones et al., 2012). While studies examining our perceptions of 3D facial volume and surfaces are relatively few, they suggest potential for future behavioral and neuroimaging studies. Further examining these 3D properties of faces using applications, such as VR or stereoscopic stimuli, will not only inform visual scientists, but also contribute knowledge of how facial perceptions are achieved in social, affective and cognitive psychology. For example, 3D models with volume, symmetry, curvilinear edges, shadows, texture, pigmentation, reflectance and illumination when systematically controlled may influence our perceptions of identity, gender, ethnicity, age, health, beauty, personality and kinship.

### The Influence of 3D Depth in Building Facial Perception

Depth is a visual cue in 2D imagery and digital screens, that creates the illusion of having linear perspective. In other words, depth cues refer to all visual features which gives humans the ability to perceive that objects are at a distance from us. When viewing a two-dimensional screen, this requires creating an illusion of depth perception from a flat surface. Without being explicitly a 3D object, depth perception is still recovered or perceived from two-dimensional flat screens, through visual cues such as stereopsis or binocular disparity, linear perspective or alignment, convergence, object constancy, illumination, shadows and textural gradients (Hallinan et al., 1999; Vishwanath and Kowler, 2004; Häkkinen et al., 2008; Lambooij et al., 2011). The illusion of more realistic or naturalistic depth, is often what separates stimuli from being considered 2D, such as we see in traditional photographs, or appearing 3D with applications such as VR (Blascovich et al., 2002; Bailenson et al., 2004) or stereoscopic glasses and monitors (Vishwanath and Kowler, 2004; Nguyen and Clifford, 2019). However, to date the exact similarities and differences between how we utilize depth perception when viewing 2D facial imagery and 3D modeled faces remains unresolved.

Several preliminary studies have investigated how depth cues influence facial recognition with 3D models presented on two-dimensional screens, however, findings so far have generally been mixed (Chang et al., 2005; Farivar et al., 2009; Dehmoobadsharifabadi and Farivar, 2016). For example, Dehmoobadsharifabadi and Farivar (2016) demonstrate that a facial identity can be recognized significantly above-chance with only one depth cue presented within 3D faces; either stereopsis, texture gradients, structure-from-motion and binocular disparity cues. While use a similar method, but identify that shading aids accurate identity recognition of 3D facial models more so than other depth cues. In eye-tracking studies, Chelnokova and Laeng (2011) demonstrate 3D stereoscopic faces alter eye-gaze attention, directing attention toward the nose and chin, more so than the eyes; an effect not commonly reported with typical 2D photographs of faces (Chelnokova and Laeng, 2011). Atabaki et al. (2015) further demonstrate there is no eye-gaze pattern or angular differences when perceiving a real human or 3D stereoscopic avatar, suggesting that viewing a stereoscopic face on a screen produces comparable attention strategies to viewing physical humans. Neuroimaging studies present evidence of increased dorsal-stream engagement when viewing 3D stimuli containing depth cues, for example stereopsis cues and structure-from motion-cues (Zeki et al., 1991; Tootell et al., 1995; Heeger et al., 1999; Backus et al., 2001; Tsao et al., 2003; Kamitani and Tong, 2006; Freud et al., 2018b). Depth added to unfamiliar faces is largely attributed to increasing both dorsal-ventral stream engagement, when presented with motion (Farivar, 2009). Overall, these initial studies infer that depth cues presented with 3D models and applications, may impact our psychophysical perception, eye-tracking and neural strategies. Comparatively, conflicting studies have also demonstrated no psychophysical improvement when comparing 2D and 3D stereoscopic faces under different task conditions, such as when poses of the face change (Hong et al., 2006) or for unfamiliar faces (Hancock et al., 2000). Liu and Ward (2006) for example, found 3D (stereo) and 2D (without stereo) produce similar recognition, suggesting that depth cues must be presented in the correct combination (Liu and Ward, 2006). Overall, this preliminary evidence suggests both similarities and differences may exist in how we perceive depth from matched 3D and 2D stimuli. Future research therefore, may benefit from directly comparing matched 2D-3D facial stimuli, as presented by Sadhya and Singh (2019) or by implementing 3D depth with VR or stereoscopic vision (Blascovich et al., 2002; Bailenson et al., 2004).

Concluding this section, depth cues such as texture and shading gradients, shape-from-motion and stereoscopic vision or binocular disparity cues, may be beneficial to identity recognition for unfamiliar faces, as well as producing similar eye-gaze attentional strategies as viewing real-life humans (Chelnokova and Laeng, 2011; Dehmoobadsharifabadi and Farivar, 2016). However, there may be no differences in depth perception between viewing 3D facial models and 2D imagery (Hancock et al., 2000; Hong et al., 2006). Depth cues presented by 3D models on-screen, may also underlie reproduction of natural eye gaze patterns toward more structural features, that would typically occur in a physical human–human interaction, and which is limited by 2D stimuli (Vishwanath and Kowler, 2004; Chelnokova and Laeng, 2011; Atabaki et al., 2015). Adding the third-dimension of depth to a facial stimulus, alongside motion, may increase both dorsal and ventral stream engagement (Farivar et al., 2009). Overall, there remains unresolved areas for future research in how depth cues of 3D and 2D models are perceived. Future research can ultimately benefit by determining how important each of these depth cues are in our facial perceptions of both 3D stimuli compared to 2D matched-stimuli (Sadhya and Singh, 2019) and by utilizing 3D models presented in VR or stereoscopic vision (Blascovich et al., 2002; Bailenson et al., 2004; Chelnokova and Laeng, 2011).

## What is the 4D Temporal Pattern of the Human Face?

### The Influence of 4D Dynamic Viewpoint Changes in Building Facial Perception

**FIGURE 6:**
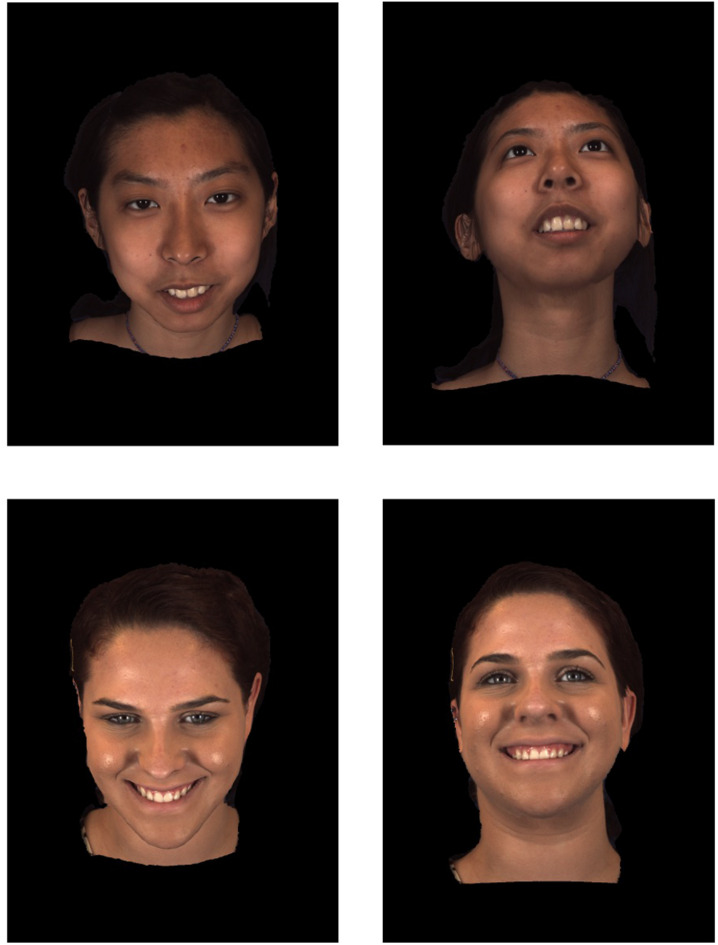
Changing facial viewpoint along a 3D axis with upper and lower head tilts (The images pictured above were reproduced, modified and adapted from the facial database originally developed by Zhang et al. (2013, 2014). Copyright 2013–2017, The Research Foundation for the State University of New York and University of Pittsburgh of the Commonwealth System of Higher Education. All Right Reserved. BP4D-Spontaneous Database, as of: 26/03/2018.

Another critical contribution of 3D facial models to understanding facial perceptions, is the ability to present faces at multiple viewpoints due to their dynamical, moving nature. Faces are highly dynamical objects which move freely in space. Thus, to examine the face as a realistic perceptual object on-screen, it must be modeled with unconstrained motion. Unconstrained motion on-screen, simply replicates the natural dynamic movements and changes in viewpoint as a person moves their head. From the observers view- This encompasses changes to our viewpoint through rotation, position, orientation, visual angle, distance, and occlusion. Similarly, this effect can be reproduced on-screen to examine facial perception with different viewpoints of the same face. One of the greatest contributions 3D face databases offer is the inclusion of unconstrained head motion within space (for some examples see Figure 6 MPI; Kaulard et al., 2012; *BP-4D-S*; Zhang et al., 2014).

Conventionally, viewpoint changes have been highly restricted in experimental research in the past. In the literature, two-dimensional faces are consistently presented at one fixed-viewpoint, which is incongruent with our daily life experience (Trebicky et al., 2018). This viewpoint is termed the “*central-frontal plane*,” where a face is positioned directly facing the camera. Indeed, the reasoning behind this decision may be sound. Incremental shifts in camera angle away from the “*central-frontal*” viewpoint of a 2D face, leads to incrementally poorer recognition performance (Lee et al., 2006). Similarly, psychophysical evidence indicates that 2D faces with a different or novel viewpoint, suffer a cost to reaction time and sensitivity (Shepard and Metzler, 1971; Bulthoff and Edelman, 1992; Hill et al., 1997; Wallraven et al., 2002). Of course, where repetitive learning or familiarity with a face-viewpoint is gained (Bulthoff and Edelman, 1992; Logothetis and Pauls, 1995; Tarr, 1995) or when experimental task demands are changed (Vanrie et al., 2001; Foster and Gilson, 2002) improvement can occur.

2D databases are available, where facial viewpoint is systematically changed in orientation or visual angle (*UT Dallas*; O’Toole et al., 2005; *AD-FES*; Van Der Schalk et al., 2011; *GEMEP Core set*; Bänziger et al., 2012; *MMI*; Valstar and Pantic, 2010; *MPI bio*; Kleiner et al., 2004) or alternatively through capturing unconstrained head movement (FG-NET FEEDtum; Wallhoff, 2004). 4D facial databases containing unconstrained head movement in a three-dimensional space have also been developed. For example, see the MPI; Kaulard et al., 2012 or *BP-4D-S*; Zhang et al., 2014. Psychophysical investigation using 3D stimuli has identified viewpoints or angles that may be beneficial for facial identification. For example, a decline in accuracy and efficiency in 3D facial recognition is observed, where viewpoint rotations occur along yaw, pitch and roll axes for 3D simulated stimuli (Favelle et al., 2011, 2017). Optimal viewpoints of unfamiliar faces, by contrast, are gained by presentation at both a frontal plane or yaw rotation (Favelle and Palmisano, 2018). Interestingly, recognition of 3D models in one study does not appear affected by viewing the asymmetrical right or left sides of the face, through yaw and roll rotations (Favelle et al., 2011, 2017).

Another study of 3D models in a VR environment, has suggested that the amount of facial information available to the visual system is the factor which most impacts recognition (Bülthoff et al., 2018). For instance, occlusion of prominent facial features or the holistic configuration of features, will reduce performance significantly in a VR environment. Consequently, pitch rotations impact recognition greatly (Bülthoff et al., 2018) – an upward pitch will produce poorer performance followed by downward pitch occlusions of 3D face stimuli (Van der Linde and Watson, 2010; Favelle et al., 2011, 2017). Roll rotations produce the greatest accuracy overall, simply because they do not contain *any* occlusion of facial features, with little disruption to the facial configuration information detected by the visual system (Favelle et al., 2011). Contrastingly, Wallis and Bülthoff (2001) used 3D head models to show that identity is not recognized well, if the identity changes as the head rotates in a temporally smooth sequence suggesting we have a strong temporal association of facial identity from memory. Thus, it appears that 3D faces with viewpoint changes may both enhance or decrease our recognition performance depending on the task conditions, with substantial contributions to the theories of featural or configural facial processing being made.

In another study, as the viewing angle of 3D model faces changed, information regarding dominance, submission and emotions displayed by another person was gained. More precisely, Mignault and Chaudhuri (2003) describe how a 3D viewpoint of a raised head tilt or upright pitch, is associated with increased personality trait-attributions of dominance and superiority; alongside emotional states of increased contempt, pride and happiness. Contrastingly, a downward head tilt or pitch implies to the viewer increased personality-attribution of submissiveness; alongside emotional states of increased sadness, inferiority, shame, embarrassment, guilt, humiliation and respect. By using 3D dynamical facial models which naturally change viewpoint and position in space, these initial studies have identified how personality and emotion perception can be impacted with viewpoint changes of the head.

Neuroimaging evidence suggests that 2D facial processing relies upon neurons sensitive to changes in viewpoint, in the Superior Temporal Sulcus (STS) and Infero-Temporal Cortex (IT). Unimodal function or exclusive firing for changes in viewpoint is demonstrated in these areas (Perrett et al., 1985, 1992; Oram and Perrett, 1992). For example, significant decrease in firing rates of these regions is observed when facial viewpoint is changed from the “*central-frontal*” plane, such as left and right profile or anterior positions (Logothetis and Pauls, 1995). These detected neurons groups may be responsible for the psychophysical findings of facial viewpoint, as discussed above. More broadly, Pourtois et al. (2005) found the Temporal cortex will respond to viewpoint changes, while the *FFA* does not respond to changes in viewer angle. To pursue this question in three dimensions, Ewbank and Andrews (2008) presented both familiar and unfamiliar 3D faces. When viewing rotation or orientation changes, face-selective voxels required more recovery from adaption (Grill-Spector et al., 1999; Andrews and Ewbank, 2004). Both emotion and viewpoint changes in 3D face stimuli did however, activate FFA (Grill-Spector et al., 1999; Andrews and Ewbank, 2004). While shape or size dimensions did not appear to matter, the FFA appeared to activate category-specific information of viewpoint change. These results are consistent with single-cell recording data of the primate infero-temporal cortex (IT) or the superior temporal sulcus (STS) and fMRI studies with human and primate subjects, which have independently studied the effects of size and viewpoint changes (Perrett et al., 1985, 1992; Sáry et al., 1993; Lueschow et al., 1994; Logothetis and Pauls, 1995; Wang et al., 1996; de Beeck et al., 2001; Pourtois et al., 2005).

In summation, 3D and 4D models have already made a substantial contribution to facial research pertaining to viewpoint change, for both familiar and unfamiliar faces (Wallis and Bülthoff, 2001; Favelle et al., 2011, 2017; Favelle and Palmisano, 2018). Renewed potential for investigations into the featural and configural theories of facial processing, has also been addressed by occlusions through viewpoint change (Van der Linde and Watson, 2010; Favelle et al., 2011, 2017; Bülthoff et al., 2018). Initial investigations into viewpoint-dependent face regions (FFA) in the brain are emerging (Grill-Spector et al., 1999; Andrews and Ewbank, 2004) although require more extensive analysis. Finally, naturalistic, unconstrained viewpoint changes provided by 4D dynamical facial modeling, may be most beneficial in understanding how we perceive emotional state and personality traits of others (Mignault and Chaudhuri, 2003).

### The Influence of 4D Dynamic Movement in Building Facial Perception

Historically, facial literature has fixated on static, 2D imagery in experimental design; that is, on-screen stimuli without movement (Kamachi et al., 2013). Overall, inconsistent findings in the literature have been reported for facial recognition relating to 2D dynamic or moving faces that were recorded with 2D capture- namely width and height (for a review see Kätsyri, 2006; Fiorentini and Viviani, 2011; Alves, 2013; Krumhuber et al., 2017). Some psychophysical studies have revealed an advantage in identification, when 2D facial motion is displayed, compared to static counter-parts (Pike et al., 1997; Wehrle et al., 2000; Knappmeyer et al., 2003; Ambadar et al., 2005; Cunningham and Wallraven, 2009) while others have not (Fiorentini and Viviani, 2011; O’Toole et al., 2011). Another significant contribution of 3D models to the facial research community therefore, is the progression of databases into four dimensions (4D). In other words, facial stimuli can now be presented with the additional dimension of dynamic movement to a 3D model. This progression is ultimately necessary when we consider that human faces are “*highly dynamical*” by nature or rarely “*still*” objects and yet are also three-dimensional. Indeed, precise variation in facial motion displayed in three dimensions, allows us to portray and communicate a wealth of information as a social species (Jack and Schyns, 2017).

Communication, or our wide-ranging emotions, mental states and speech, is conveyed to other humans principally through two forms of movement which are built into 3D digitized models. Facial movement can be modeled as rigid and non-rigid motion (for review see Smeets et al., 2012; Chrysos et al., 2018). Evidence suggests anatomically accurate rigid and non-rigid facial movements are essential to facial perception (Trautmann et al., 2009; Johnston et al., 2013) and is achievable computationally (Zhang et al., 2003; Zhang and Fisher, 2019). Deformable facial modeling, relates to movement we produce through voluntary, striated musculature or facial muscles. For example, when we talk, raise our eyebrows or smile, we activate this musculature system. Rigid head modeling, relates to the constrained-motion produced by the head, which is controlled through the neck musculature. An example of rigid motion is when we change the posture of our head, such as when shaking our head in disagreement or nodding in agreement. Both deformable modeling and rigid head modeling help reproduce naturalistic movements on-screen. Briefly, some stand-out examples of facial databases with motion include the *BU4D-S* (Zhang et al., 2014) and the *D3DFACS* (Cosker et al., 2011). However, also see (Wang et al., 2004; Vlasic, 2005; Gong et al., 2009; Weise et al., 2009; Garrido et al., 2013; Shi et al., 2014; Suwajanakorn et al., 2014; Zhang et al., 2014; Cao et al., 2015; Thies, 2015; Jeni et al., 2017). Comprehensive reviews comparing the static (3D) and dynamic (4D) datasets are also available, as evaluated by Sandbach et al. (2012) and Smeets et al. (2012). However, a recent shift toward dynamic, moving facial databases has also been observed in the 2D literature and is worth mentioning (*for a review see* Krumhuber et al., 2013; Krumhuber et al., 2017).

**FIGURE 7:**
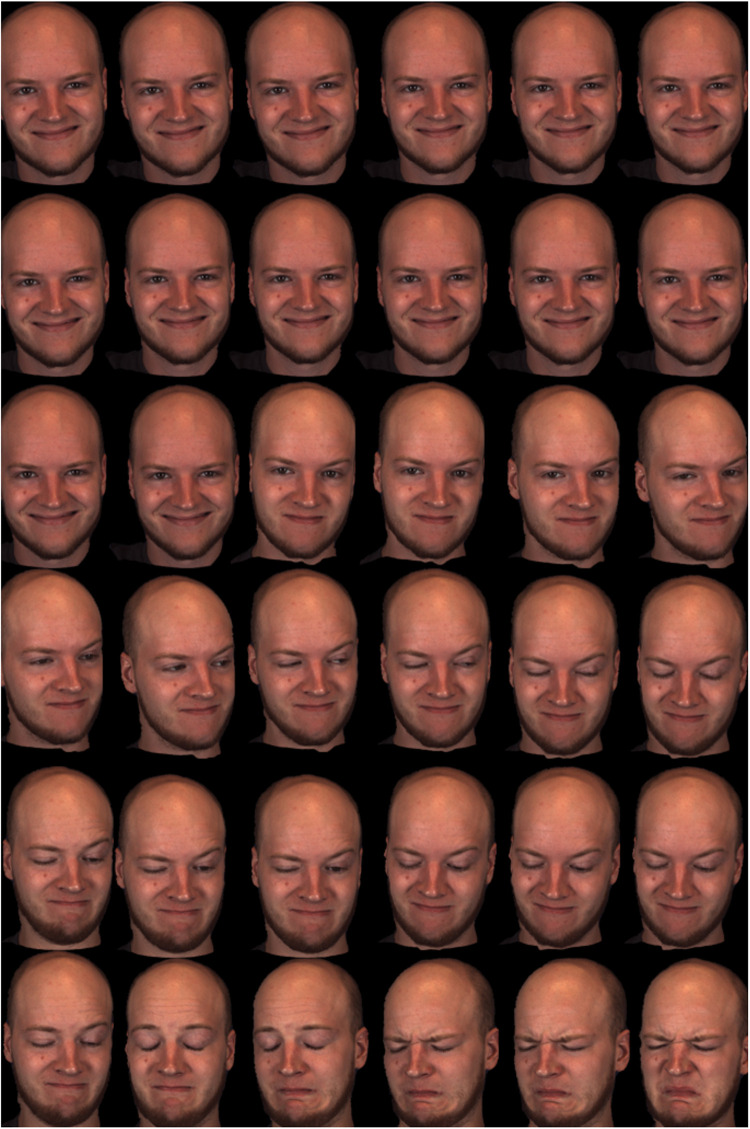
Video-recorded dynamic 4D facial stimulus captured as still frame-by-frame images, displaying happy and disgusted FACS coded emotion (The images pictured above were reproduced, modified and adapted from the facial database originally developed by Zhang et al. (2013, 2014). Copyright 2013–2017, The Research Foundation for the State University of New York and University of Pittsburgh of the Commonwealth System of Higher Education. All Right Reserved. BP4D-Spontaneous Database, as of: 26/03/2018. This exemplar presents the frame-by-frame stills from a video-recording of a 4D face, moving from a happy expression to a disgusted expression.

The emerging field of 4D facial research is therefore likely to offer answers into how naturalistic social, cognitive and affective perceptions are achieved when observing facial motion. Deformable movement is the more popular facial research area, encompassing studies of speech, language and emotional expressions. Deformable movement of our facial musculature in the literature is defined popularly by the Facial Action Coding System (Ekman and Friesen, 1978; Ekmans and Rosenberg, 1997). FACS is a highly cited, objective system of measuring facial movement activated by groups of facial muscles, defined as “action units.” (Ekman and Friesen, 1978; Ekmans and Rosenberg, 1997 44 action units, the FACS system groups facial muscles which activate collectively. These activations are ultimately what produce deformable expression and speech output. For example, an emotion pattern such as a disgusted response, can be quantified into an action unit or series of muscle activations (AUs), such as a “grimace” of the mouth and “screwing up” of the eyes. Exemplars of different FACS coded muscle activations, illustrating smiles, frowns and grimaces of deformable facial features are displayed in Figure 7.

Recently, Savran et al. (2012) presented a detailed review to compare how the FACS system aids perception of 2D and 3D faces, suggesting emotion recognition with the FACS system is superior in 3D faces, particularly with low intensity emotions. While the FACS system has been extensively used with 2D dynamic faces displaying emotional expressions, for example, 2D images (Pantic and Rothkrantz, 2004; Cohn and De la Torre, 2014; Baltrušaitis et al., 2015) and 2D dynamic videos (Donato et al., 1999; Pantic and Rothkrantz, 2004; Bartlett et al., 2006) this study indicates 3D/4D dynamic faces may be more important for low intensity emotions. The FACS system has likewise achieved action unit detection in 3D facial videos or scans (Cosker et al., 2011) for successful emotion detection (Sun et al., 2008; Tsalakanidou and Malassiotis, 2010; Berretti et al., 2013; Reale et al., 2013; Tulyakov et al., 2015; Danelakis et al., 2018); and even more socially related morphologies such as dominance, trustworthiness and attractiveness (Gill et al., 2014) or the influences of ethnicity and gender on how we display emotional expressions differently from one another (Jack et al., 2014; Jandova and Urbanova, 2018).

Deformable modeling has made a further advancement in facial stimuli design, by digitizing naturalistic, spontaneous movements, rather than the synthesized, artificial movements produced in the past (Wehrle et al., 2000; Tcherkassof et al., 2007). For example, high classification accuracy using the 3D FACS system has been demonstrated for spontaneous emotions displayed in these databases (96%; Tarnowski et al., 2017). Spontaneous deformation provides computer scientists the ability to track anatomically accurate emotional displays from humans in-situ (Bartlett et al., 2006; Zhang et al., 2014). This is of consequence to emotion and language research, as motion or timing of deformable changes, is considered important (Bassili, 1978; Ekman, 1982; Wehrle et al., 2000; Kamachi et al., 2013). This is exemplified in the case of “*false smiles*.” For example, while the FACS activated in any smile is highly similar, it is the *timing* of activation which reveals the difference between a deceptive smile and a smile of genuine happiness, as can the consistency between the eye and mouth FACS (Ekman, 1982; Cohn and Schmidt, 2003; Guo et al., 2018). 4D faces have also helped delineate *when* we can evaluate an emotional expression. For example, most expressions begin the same, with the 6 basic emotional expressions becoming revealed temporally later in Western Caucasians, as shown through the FACS system (Jack et al., 2014). More culturally specific, East Asians have demonstrated more overlap and less specificity in the FACS displaying surprise, fear, disgust and anger, compared to Western Caucasians (Jack et al., 2014). Overall, deformable modeling of 4D faces combined with 3D FACS, offer researchers a promising methodology of speech, language and emotion which use deformable facial musculature.

Albeit less studied, rigid modeling of 4D face datasets is also of key importance to include in dynamic stimuli presentation. Rigid head movement is defined as that constrained by the neck, involving changes to the position or posture of the head. Rigid motion in the literature is defined and measured along 3D axes, including changes in head tilt, pitch, yaw and roll. Using 3D neutral facial models, emotional states or personality traits associated with head tilt include a downward head angle as guilt, sadness, embarrassment, gratitude, fear, surprise, shame, guilt as submissive displays upward head angle as pride, contempt, and scorn as dominance (Mignault and Chaudhuri, 2003). For example, rigid head motion alone has demonstrated the ability to determine an individuals’ identity (Hill and Johnston, 2001). 3D head posture alone, with neutral expression, indicates a wide array of information (Mignault and Chaudhuri, 2003), including gender-based displays of dominance and aggression, or alternatively submission. Many traits are perceived through head tilt; including a lowered head indicating sadness, inferiority and submissiveness (Otta et al., 1994). Overall, these initial studies of unconstrained movement, while relatively rare, have provided methodologies whereby 4D faces can be used to improve studies of language, emotion and personality traits, alongside social factors such as gender and ethnicity. Thus, it is likely a growing number of studies in future will utilize facial stimuli with unconstrained head motion, as presented by deformable and rigid head modeling. Overall, both rigid and deformable modeling of 4D faces combined with the 3D FACS system, offer psychologists a promising methodology for all areas of speech, language and emotion research.

## Recommendations

In this review, we discuss the nature of 4D face stimuli that are modeled over space and time. The review introduces the available 3D/4D face models and databases for researchers, while considering how these are captured and reconstructed for experimental design. Finally, we review studies which incorporate 3D spatial and 4D temporal properties to reveal how volume, surfaces, depth, viewpoint and movement affect our perceptions of others. In sum, we recommend the implementation of 4D stimulus models built from real-life human faces with anatomically accurate spatial and temporal dimensions, to resolve many unanswered questions of facial perception.

Future research in the vision sciences can ultimately benefit by determining how important 3D structural and depth properties are in our facial perceptions of 3D models compared to 2D matched-stimuli (Sadhya and Singh, 2019). Secondly, at the start of this revolution, we will begin to understand how facial perception is realized in increasingly naturalistic settings, which capture and reproduce the complex interaction of 3D volume, surface, depth, viewpoint and movement properties (Bulthoff and Edelman, 1992; Bailenson and Yee, 2006; Stolz et al., 2019; Lamberti et al., 2020). For example, 3D/4D facial models enable complex visual cues and 3D depth within naturalistic social environments, once integrated with applications such as VR and stereoscopic technology (Bailenson et al., 2004; Stolz et al., 2019; Lamberti et al., 2020). By assimilating complex models of face stimuli into future research, we can resolve long-standing questions about how we identify familiar compared to unfamiliar faces, especially from different viewpoints (Wallis and Bülthoff, 2001; Abudarham et al., 2019; Zhan et al., 2019). We may also improve understanding of how we identify social characteristics of individuals; such as gender, ethnicity or personality traits. We can better explore social perceptions with 3D facial features and surfaces by investigating many unresolved questions, such as what the nose or eyes reveal about ethnicity and gender? (Lv et al., 2020) or how do we identify race, health and age from skin surfaces? (Jones et al., 2012). Likewise, we can examine the decoding of more complicated emotional expressions, speech and language with 3D FACS over time (Lu et al., 2006; Jack et al., 2014). Finally, we may better understand the reasons why emotional expressions, inversion or movement, present challenges for specific clinical subpopulations during facial perception tasks, such as in Autism Spectrum Disorder (Weigelt et al., 2012; Anzalone et al., 2014) or Schizophrenia (Onitsuka et al., 2006; Wang et al., 2007; Marosi et al., 2019).

To meet the challenges of integrating complex 4D face models in psychological methodology, more complex computational methods of analysis will be required. It is recommended that future experiments utilize multivariate time-resolved methods of analysis. Thus, as a facial stimulus evolves over time, methods of analysis should simultaneously capture this dynamical information. Existing methods such as psychophysical behavioral measures (eye-tracking, reaction-time, inspection time, accuracy) or neuroimaging measures (decoding, multivariate pattern analysis or time-frequency analysis) are recommended.

## Conclusion

In conclusion, this in-depth review highlights the effects of presenting on-screen facial stimuli with both 3D visual properties (volume, surface and depth) and 4D dynamical properties (viewpoint, movement). We conclude that *the human face is inherently defined as a 4D dynamic* perceptual object. Ignoring this definition in experimental stimuli remains a widespread issue which confounds our understanding of naturalistic human facial perception is performed. To date, the emerging use of 4D dynamic models in facial research has so far revealed an amazing richness in facial perceptions of identity, gender, ethnicity, personality traits, speech and emotional expressions.

## Author Contributions

AB wrote the manuscript including synthesis of literature search, performed manuscript writing and editing. DC performed the editing. Both authors contributed to the article and approved the submitted version.

## Conflict of Interest

The authors declare that the research was conducted in the absence of any commercial or financial relationships that could be construed as a potential conflict of interest.
